# Transcriptome Analysis Identifies Key Candidate Genes Mediating Purple Ovary Coloration in Asiatic Hybrid Lilies

**DOI:** 10.3390/ijms17111881

**Published:** 2016-11-20

**Authors:** Leifeng Xu, Panpan Yang, Suxia Yuan, Yayan Feng, Hua Xu, Yuwei Cao, Jun Ming

**Affiliations:** 1 Institute of Vegetables and Flowers, Chinese Academy of Agricultural Sciences, Beijing 100081, China; xlf511@126.com (L.X.); yangpanpan@126.com (P.Y.); ysxia8@126.com (S.Y.); 13161164012@163.com (Y.F.); ahxuhua@163.com (H.X.); 13067739520@163.com (Y.C.); 2 College of Landscape Architecture, Nanjing Forestry University, Nanjing 210037, China

**Keywords:** *Lilium* spp., anthocyanins, purple ovaries, transcriptome, transcriptional network

## Abstract

Lily tepals have a short lifespan. Once the tepals senesce, the ornamental value of the flower is lost. Some cultivars have attractive purple ovaries and fruits which greatly enhance the ornamental value of Asiatic hybrid lilies. However, little is known about the molecular mechanisms of anthocyanin biosynthesis in Asiatic hybrid lily ovaries. To investigate the transcriptional network that governs purple ovary coloration in Asiatic hybrid lilies, we obtained transcriptome data from green ovaries (S1) and purple ovaries (S2) of Asiatic “Tiny Padhye”. Comparative transcriptome analysis revealed 4228 differentially expressed genes. Differential expression analysis revealed that ten unigenes including four *CHS* genes, one *CHI* gene, one *F3H* gene, one *F3′H* gene, one *DFR* gene, one *UFGT* gene, and one *3RT* gene were significantly up-regulated in purple ovaries. One *MYB* gene, *LhMYB12-Lat*, was identified as a key transcription factor determining the distribution of anthocyanins in Asiatic hybrid lily ovaries. Further qPCR results showed unigenes related to anthocyanin biosynthesis were highly expressed in purple ovaries of three purple-ovaried Asiatic hybrid lilies at stages 2 and 3, while they showed an extremely low level of expression in ovaries of three green-ovaried Asiatic hybrid lilies during all developmental stages. In addition, shading treatment significantly decreased pigment accumulation by suppressing the expression of several unigenes related to anthocyanin biosynthesis in ovaries of Asiatic “Tiny Padhye”. Lastly, a total of 15,048 Simple Sequence Repeats (SSRs) were identified in 13,710 sequences, and primer pairs for SSRs were designed. The results could further our understanding of the molecular mechanisms of anthocyanin biosynthesis in Asiatic hybrid lily ovaries.

## 1. Introduction

Lily (*Lilium* spp.) is one of the most important ornamental plant species worldwide, owing to its large flowers, which have diverse tepal colors, unique shapes, and attractive floral scents. However, lily tepals have a short lifespan, which ends with wilting or abscission. Lily inflorescence longevity is approximately 2 weeks, and individual flower longevity is limited to approximately 1 week. Once the tepals senesce, the ornamental value of the flower is lost. However, some cultivars have purple ovaries, which develop into attractive purple fruits. These fruits maintain their color for approximately 2 months, which greatly enhances the ornamental value of these hybrid lilies. This ornamental trait can be developed for container-grown and outdoor-planted lily cultivars, and we believe that it has great potential to increase the ornamental value of hybrid lilies.

Anthocyanins, a class of flavonoid compounds, are the main pigments in flowers and fruits where they act as visual cues for pollinators and seed distributors [1]. Large variations in the intensity, hue, and patterning of anthocyanins are observed among angiosperm flowers [2]. In floricultural crops, these variations are one of the most important factors determining the market value of flowers. The biosynthetic pathway of anthocyanins has been studied extensively in different plant species and is one of the best-known pathways of specialized metabolism [3,4]. The genes in the anthocyanin biosynthetic include chalcone synthase (*CHS*), chalcone isomerase (*CHI*), flavonoid 3-hydroxylase (*F3H*), flavonoid 3′-hydroxylase (*F3'H*), flavonoid 3′5′ hydroxylase (*F3′5′H*), dihydroflavonol 4-reductase (*DFR*), anthyocyanidin synthase (*ANS*), and UDP-glucose:flavonoid 3-*O*-glucosyltransferase (*UFGT*) [3,5]. The expression of these biosynthetic genes is mainly controlled at the transcriptional level and is regulated by a complex consisting of R2R3-MYB, basic helix–loop–helix (bHLH), and WD-repeat transcription factors [5,6]. Among these transcription factors, the R2R3-MYB transcription factors play a major role in determining the spatio-temporal expression of anthocyanin biosynthetic genes.

There have been some reports on the molecular mechanisms of flower colors and color patterning in Asiatic hybrid lilies in recent years. Three *CHS* genes (*LhCHSA*, *LhCHSB*, and *LhCHSC*) and one *DFR* gene (*LhDFR*) were isolated from Asiatic “Montreux” [7]. The full-length cDNA sequences of *LhPAL*, *LhF3H*, *LhF3′H*, and *LhANS* were isolated from the tepals of Asiatic lily “Montreux” [8], and two *R2R3-MYB* transcription factors, *LhMYB6* and *LhMYB12*, were also isolated from Asiatic “Montreux” [9]. LhMYB12 was shown to directly bind to the promoters of *LhCHSA* and *LhDFR* to activate their expression [8]. In another study, *LhMYB12-Lat*, which contributes to the splatter pigmentation of the Tango Series cultivars of Asiatic hybrid lilies, was isolated from anthocyanin-accumulating tepals of Asiatic “Latvia” [2]. In addition to the large variations in flower colors and color patterning, Asiatic hybrids also show variations in ovary colors. Most Asiatic hybrids have green ovaries (e.g., “Yellow Pixels”), but a few (e.g., “Tiny Padhye”) have purple ones. However, little is known about the molecular mechanisms of purple ovary coloration in *Lilium* spp.

In the present study, the transcriptomes of stage 1 (green) and stage 2 (purple) ovaries of the purple-ovaried cultivar “Tiny Padhye” were sequenced using an Illumina Hiseq 4000 sequencer. After analyzing the data, we identified some key candidate genes related to anthocyanin biosynthesis in *Lilium* ssp. These transcriptome sequences may provide a valuable genomic resource to further our understanding of the molecular mechanisms of purple ovary coloration in Asiatic hybrid lilies.

## 2. Results

### 2.1. Measurement of Anthocyanin Content

**Figure 1:**
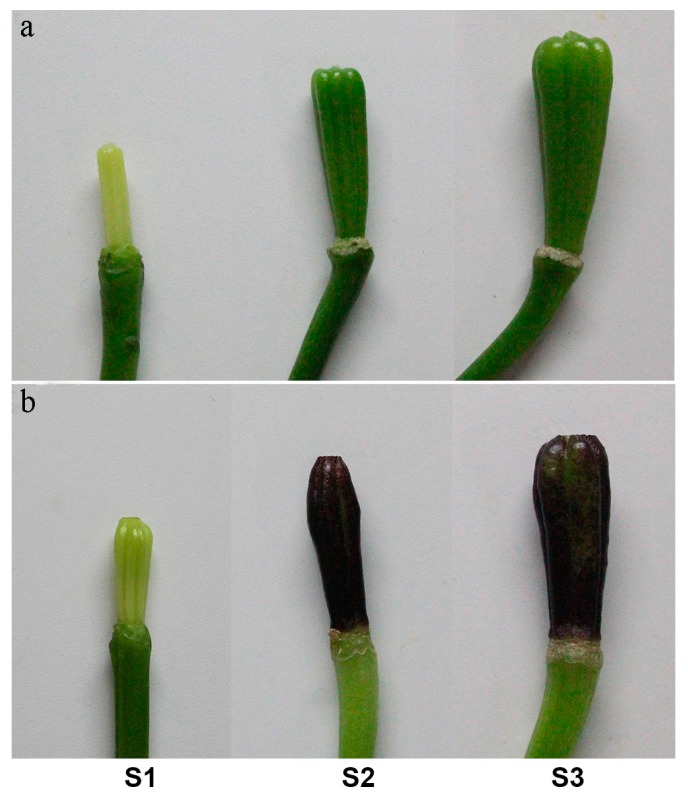
Phenotype of ovaries of Asiatic “Yellow Pixels” (**a**) and “Tiny Padhye” (**b**) at three ovary developmental stages.

**Figure 2:**
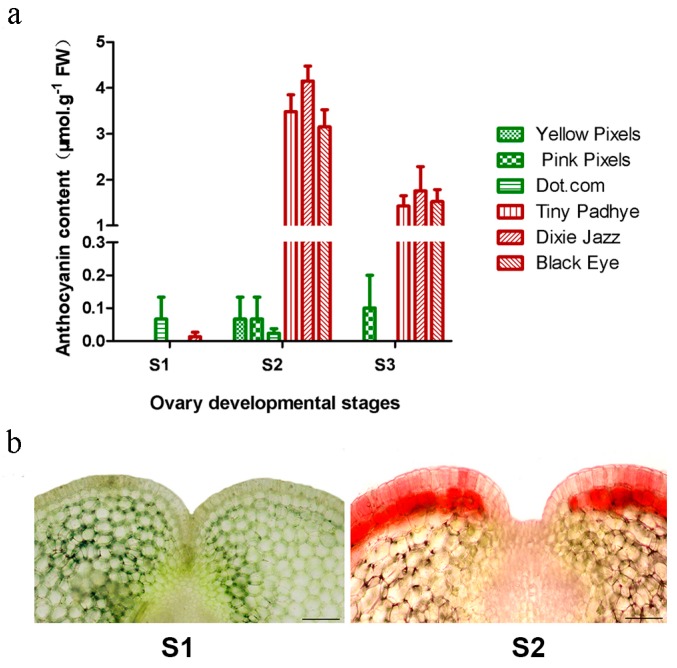
Relative anthocyanin content and cross sections of ovaries. (**a**) The relative anthocyanin content of lily ovaries at different developmental stages. Error bars show the standard error (SE) of the mean; (**b**) Cross sections of ovaries of Asiatic “Tiny Padhye” at developmental stage 1 and stage 2. Scale bars: 100 μm.

A single anthocyanin pigment (cyanidin 3-*O*-β-rutinoside) was detected in the pigmented ovaries of the cultivar “Tiny Padhye”. During the two stages (S1 and S2) of ovary development of Asiatic “Tiny Padhye”, the ovaries underwent a rapid change in colors from green to purple (Figure 1b). This change was due to the increase of anthocyanin content from 0.05 to 4.75 μmol·g^−1^ fresh weight (FW) (Figure 2a). Microscopic examination of fresh hand-cut sections of ovaries of Asiatic “Tiny Padhye” showed that anthocyanins accumulated in both epidermal and sub-epidermal layers in purple ovaries (S2), but not in green ones (S1) (Figure 2b).

### 2.2. Sequencing and Sequence Assembly

The transcriptomes of green ovaries (S1) and purple ovaries (S2) of Asiatic “Tiny Padhye” were obtained by Illumina technology. Approximately 157 million and 145 million raw reads were generated from green ovaries and purple ovaries, respectively. After removing low-quality sequences, adapters, and ambiguous reads, we obtained approximately 153 million (S1) and 141 million (S2) high-quality clean reads (Table S1). More than 94% of bases had a *q*-value > 20 for both samples (Table S1). All raw reads were archived at NCBI Sequence Reads Archive (SRA) under accession number SRP081210. In total, approximately 294 million clean reads were used to assemble the transcriptome data using the Trinity method.

### 2.3. Identification of Differentially Expressed Genes

**Figure 3:**
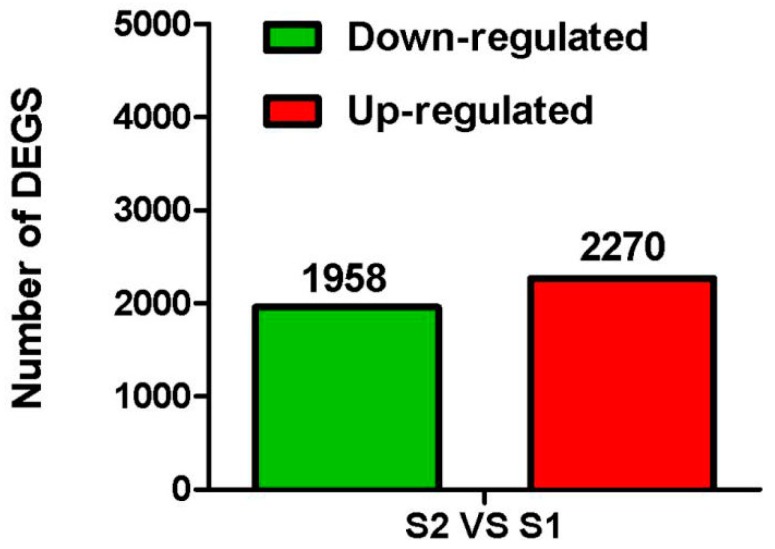
The number of up- and down-regulated unigenes in a comparison of S2 to S1.

The genes showing differences in expression between the two samples were identified and filtered. A total of 4228 unigenes were identified as being significantly differentially expressed, of which 2270 showed higher expression in purple ovaries, and 1958 showed higher expression in green ovaries (Table S2 and Figure 3).

### 2.4. Functional Analysis of Differentially Expressed Genes

**Figure 4:**
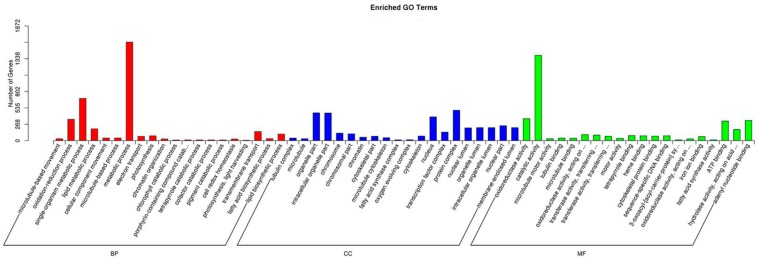
The distribution of enriched Gene Ontology (GO) term categories of differentially expressed genes (DEGs).

To further illustrate the main biological functions of the transcriptome, we conducted Gene Ontology (GO) enrichment and Kyoto Encyclopedia of Genes and Genomes (KEGG) pathway analyses. A GO enrichment analysis provides a description of gene products in terms of their associated Biological Process (BP), Cellular Component (CC), and Molecular Function (MF) [10]. The obtained differentially expressed genes (DEGs) were categorized into 60 major functional groups, and metabolic process (GO:0008152), protein complex (GO:0043234), and catalytic activity (GO:0003824) were the most highly represented GO terms in BP, CC, and MF, respectively (Figure 4).

In the KEGG pathway analysis, the unigenes were mapped to 127 pathways, and 57 unigenes were involved in the anthocyanin biosynthesis pathway (Table 1). The DEGs up-regulated in purple ovaries were mapped to 102 KEGG pathways. The DEGs up-regulated in green ovaries were mapped to 94 KEGG pathways. In total, 19 of the DEGs up-regulated in purple ovaries were involved in the anthocyanin biosynthesis pathway (Table 1 and Table 2).

### 2.5. Expression Patterns of Genes Involved in Anthocyanin Biosynthesis Pathway

**Figure 5:**
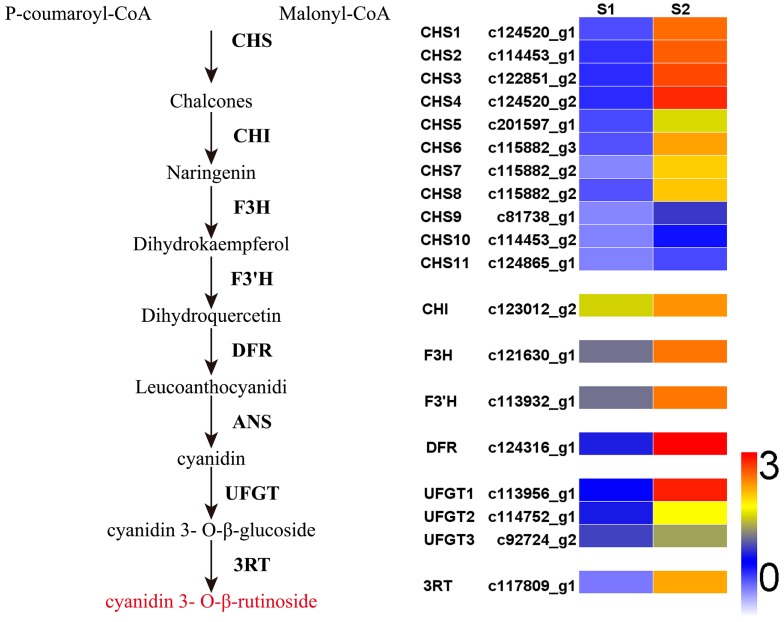
Heatmap of DEGs related to anthocyanin biosynthesis pathway. The expression pattern of each unigene was shown using different grids from blue to red, with the values representing the log_10_ (FPKM + 1) values. FPKM standed for Fragment Per Kilobase of transcript per Million mapped reads.

The anthocyanin biosynthesis pathway produced the purple anthocyanin pigment that gave the purple ovaries their color. Therefore, unigenes involved in the anthocyanin biosynthesis pathway were selected and studied in detail. We identified 57 unigenes encoding seven putative enzymes involved in anthocyanin biosynthesis from the transcriptomes. Among the 57 unigenes, 19 were DEGs. After further analysis of the expression patterns of these DEGs, we found that *LhCHS1* (c124520_g1), *LhCHS2* (c114453_g1), *LhCHS3* (c122851_g2), *LhCHS4* (c124520_g2), *LhCHI* (c123012_g2), *LhF3H* (c121630_g1), *LhF3'H* (c113932_g1), *LhDFR* (c124316_g1), *LhUFGT1* (c113956_g1), and *Lh3RT* (c117809_g1) showed significantly higher transcript levels in purple ovaries than in green ones (Table 2 and Figure 5). The expression of most of the structural genes in the anthocyanin biosynthetic pathway is coordinately regulated by a ternary complex comprising three groups of transcription factors: R2R3-MYB, bHLH, and WDR transcription factors [5]. Thus, we analyzed the expression levels of regulatory factors that potentially control anthocyanin biosynthesis. We identified 36 DEGs encoding candidate transcription factors from the DEG data, including eight unigenes encoding R2R3-MYBs, 19 encoding bHLHs, and nine encoding WD40s (Table 1). Among these transcription factor unigenes, 19 were highly expressed in purple ovaries and 17 were highly expressed in green ones (Table 1).

### 2.6. Phylogenetic Analysis of R2R3-MYB Transcription Factors

**Figure 6:**
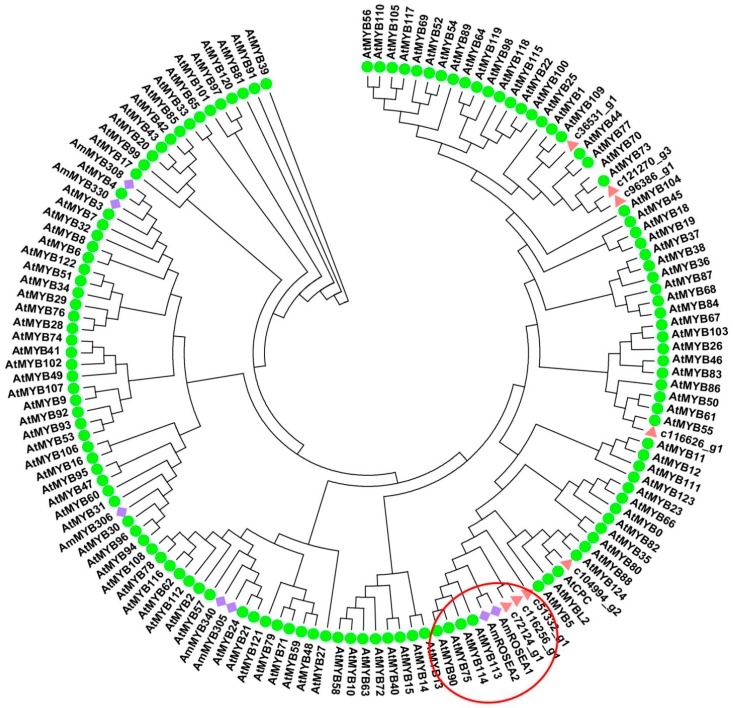
Phylogenetic relationships of selected R2R3-MYB transcription factors in plants. The GenBank accession numbers are as follows: AmMYB308 (P81393), AmMYB340 (P81396), AmMYB306 (P81392), AmMYB305 (P81391), AmROSEA1 (ABB83826), AmROSEA2 (ABB83827), AmMYB330 (P81395), and all arabidopsis R2R3-MYB proteins were retrieved from TAIR (Available online: https://www.arabidopsis.org). Plant anthocyanin-promoting R2R3-MYBs were shown in the red circle.

To study the R2R3-MYB transcription factors that regulate anthocyanin biosynthesis in ovaries, we identified eight R2R3-MYB transcription factors among the DEGs. In the phylogenetic analysis, c51332_g1, c72124_g1, and c116256_g1 were in the same cluster as plant anthocyanin-promoting R2R3-MYBs, including AmROSEA1, AmROSEA2, and AtMYB75/90/113/114 (Figure 6). The FPKM value of *LhMYB* (c51332_g1) was significantly higher in purple ovaries than in green ones (Table 2). In the dendrogram, c36531_g1, c121270_g3 and c96386_g1 were grouped together, c116626_g1 was in another clade with AtMYB55, and c104994_g2 was grouped with AtCPC (Figure 6).

### 2.7. qPCR Validation of RNA-Seq Data

**Figure 7:**
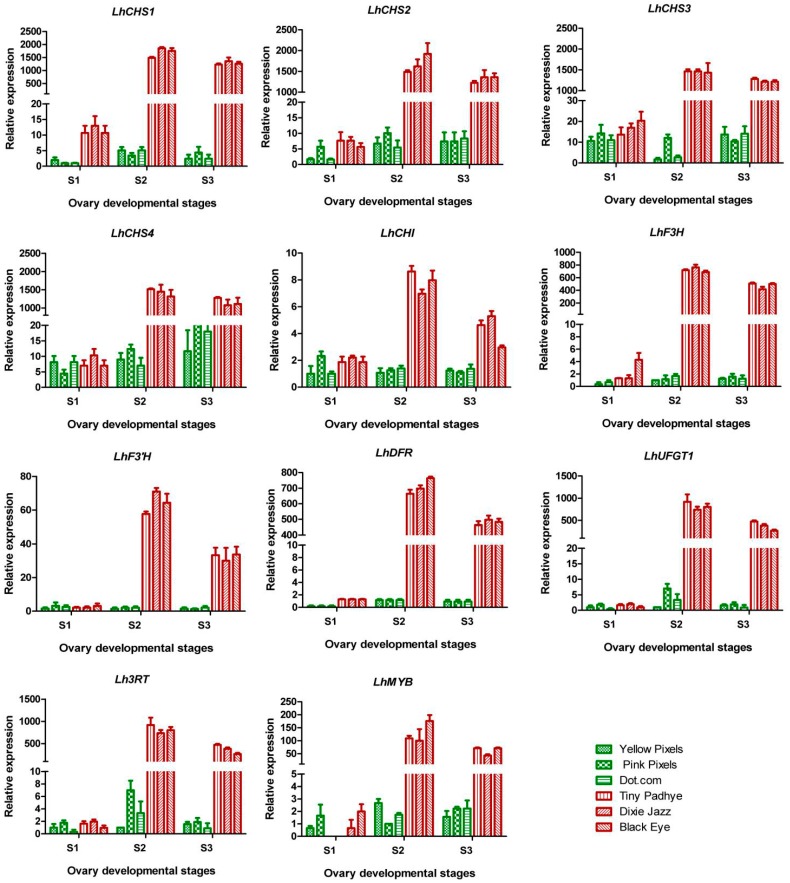
Expression profiles of *LhMYB* and candidate anthocyanin structural genes in ovaries of Asiatic “Tiny Padhye”, “Black Eye”, “Dixie Jazz”, “Dot.com”, “Pink Pixels”, and “Yellow Pixels”. Error bars show the SE of the mean.

To validate the reliability of the comparative transcriptional data and to further analyze the differences in the expression profiles between purple-ovaried and green-ovaried Asiatic hybrid lilies during the three different development stages, 11 unigenes related to anthocyanin biosynthesis (*LhCHS1*, *LhCHS2*, *LhCHS3*, *LhCHS4*, *LhCHI*, *LhF3H*, *LhF3'H*, *LhDFR*, *LhUFGT1*, *LhMYB*, and *Lh3RT*) were selected for qPCR analyses. In the three purple-ovaried cultivars, all the chosen unigenes were highly expressed at stages 2 and 3, while their transcripts were almost undetectable or showed a low level of expression at stage 1 (Figure 7). These qPCR results were consistent with those obtained from the DGE expression profiling (Table 1 and Figure 7). In the three green-ovaried cultivars, transcripts of these unigenes showed an extremely low level of expression during all developmental stages (Figure 7). These qPCR results were consistent with the accumulation of anthocyanins in these cultivars (Figure 2a and Figure 7).

### 2.8. Effect of Shading Treatment on Pigment Accumulation and Gene Expression

**Figure 8:**
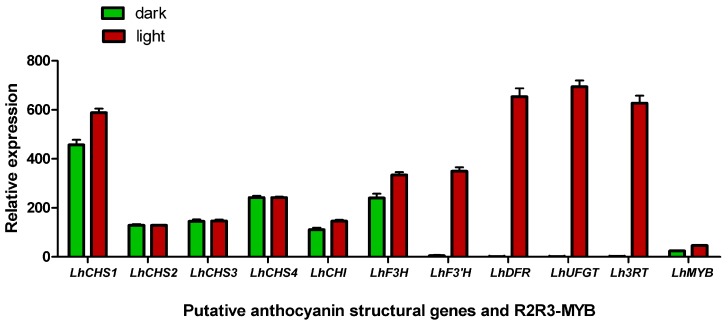
Effect of shading treatment on gene expression in ovaries of Asiatic “Tiny Padhye”. Error bars show the SE of the mean.

To investigate whether shade treatment affects pigment accumulation and gene transcription, 2.50 cm Asiatic lily “Tiny Padhye” flower buds were covered with silver paper. Relative anthocyanin levels were very low in the shaded ovaries compared with those in the control group, indicating that the shade treatment significantly decreased the relative anthocyanin content. Furthermore, qPCR showed that expression of four anthocyanin structural genes, *LhF3'H*, *LhDFR*, *LhUFGT1*, and *Lh3RT*, was high in the control ovaries, but was extremely low or undetectable in the shaded ovaries (Figure 8). *LhCHS1*, *LhCHI*, *LhF3H*, and *LhMYB* expression levels were lower in the shaded ovaries than in the control ovaries, whereas *LhCHS2*, *LhCHS3*, and *LhCHS4* expression in ovaries was not affected by the shade treatment (Figure 8).

### 2.9. Simple Sequence Repeat (SSR) Marker Identification and Characterization

In total, 15,048 SSRs were identified in 13,710 sequences (Table 3). Of these sequences, 1201 sequences contained more than one Simple Sequence Repeat (SSR) and 444 SSRs were present in the compound formation. Among the identified SSRs, the largest category of the SSRs was mononucleotide (8163, 54.2%), followed by dinucleotide (4446, 29.5%), trinucleotide (2309, 15.3%), tetranucleotide (96, 0.6%), hexanucleotide (19, 0.1%), and pentanucleotide (15, 0.1%) repeats. Using Primer 3.0 software, primer pairs for each SSR were designed (Table S4).

## 3. Discussion

### 3.1. Co-Expression of Genes Involved in Anthocyanin Biosynthesis Determines Anthocyanin Accumulation in Purple Ovaries

In this study, 57 unigenes involved in the anthocyanin biosynthetic pathway were selected and studied. To the best of our knowledge, this is the first time that all of the structural genes in the anthocyanin biosynthesis pathway have been identified in *Lilium* ssp. These data will help us to elucidate the regulatory mechanisms of anthocyanin biosynthesis in lily ovaries and tepals. There were several instances where more than one unigene was annotated as the same enzyme (Table 2), which might be because these unigenes represented different members of the same gene family or different fragments of a single transcript [11]. Among the 57 unigenes, we identified that four *CHS* genes, one *CHI* gene, one *F3H* gene, one *F3'H* gene, one *DFR* gene, one *UFGT* gene, and one *3RT* gene were significantly up-regulated in purple ovaries, compared with green ovaries. We speculate that the high co-expression of these enzymes involved in anthocyanin biosynthesis is responsible for anthocyanin accumulation in the purple ovaries. Further studies are required to determine whether changes in the transcript levels of these candidate genes are related to anthocyanin metabolism.

### 3.2. LhMYB12-Lat Is Responsible for Purple Ovaries

In recent years, several studies have characterized regulatory genes involved in red and purple fruit coloration in eudicots and monocots. In apple (*Malus × domestica*), two *MYB* genes, *MdMYB1* and *MdMYBA*, are responsible for anthocyanin biosynthesis in red-skinned apple cultivars [12,13]. Similarly, in grapevine (*Vitis vinifera*), *VvMYBA1* and *VvMYBA2* are responsible for anthocyanin biosynthesis in red-skinned cultivars [14]. *GmMYB10*, *MrMYB1*, *PcMYB1*, and *LcMYB1* are involved in the anthocyanin biosynthetic pathway in mangosteen (*Garcinia mangostana*), Chinese bayberry (*Myrica rubra*), pear (*Pyrus pyrifolia*), and litchi *(Litchi chinensis*), respectively [15,16,17,18]. In maize (*Zea mays*) seeds, anthocyanin biosynthesis is regulated by *C1* [19]. In this study, the phylogenetic analysis showed that c51332_g1, c72124_g1, and c116256_g1 were grouped into the same cluster with plant anthocyanin-promoting R2R3-MYBs, and the FPKM value of c51332-g1 was significantly higher in purple ovaries than in green ones (Table 2). In addition, the sequence of c51332_g1 was the same as that of *LhMYB12-Lat*, which contributes to the splatter pigmentation of the Tango Series cultivars of Asiatic hybrid lilies [2]. Therefore, we speculate that LhMYB12-Lat is the main anthocyanin-promoting protein that determines the distribution of anthocyanin pigments in Asiatic hybrid lily ovaries. These results indicate that, in both monocots and eudicots, *R2R3-MYB* genes have crucial roles in determining the distribution of anthocyanin pigments in fruits. Interestingly, in the Tango Series of Asiatic hybrid lily cultivars, some cultivars have green ovaries even though their tepals have splattered pigmentation due to the expression of *LhMYB12-Lat*. We suggest three possible explanations for this phenomenon. First, some negative regulators of the anthocyanin biosynthesis pathway may exist in ovaries of green-ovaried cultivars (e.g., a R2R3-MYB MYB repressor, a competitive R3-MYB repressor, or a competitive bHLH repressor). Alternatively, the expression of *LhMYB12-Lat* may be influenced by other factors such as microRNAs and/or NAC/SPL transcription factors. Last, methylation of the promoter of *LhMYB12-Lat* may affect its expression.

### 3.3. Shading Treatment Decreased Pigment Accumulation

Light is one of the important environmental factors that affect the anthocyanin biosynthesis [20]. Numerous studies have shown that light exposure can enhance the anthocyanin accumulation in fruit skin [21,22]. In addition to light intensity, light quality also influences the concentration of anthocyanins in fruits of many plant species. Anthocyanin accumulation is enhanced by ultraviolet-B light in apple [23,24,25] and red Chinese sand pear (*P. pyrifolia*) [26]. Blue light induces anthocyanin accumulation and expression of associated genes in apple fruits [27], strawberry (*Fragaria × ananassa*) fruits [28], and Chinese bayberry fruits [29]. In this study, compared with the control group, the shading treatment significantly decreased pigment accumulation by suppressing the expression of several anthocyanin structural genes and a related regulatory gene in ovaries of Asiatic “Tiny Padhye”. However, whether light quality and the wavelength of light affect anthocyanin biosynthesis in Asiatic lily ovaries remains unclear. This issue should be investigated further in future studies.

### 3.4. SSR Marker Identification and Characterization

Microsatellites or Simple Sequence Repeats (SSRs) are important for gene mapping and molecular breeding in plant research. Previously, approximate 5000 SSR markers had been reported in *Lilium* [30,31,32,33]. Considering the large and complicated lily genome, these makers are not sufficient for effective genetic mapping and marker-assisted selection breeding. Here, a total of 15,048 SSRs were identified in 13,710 sequences. The majority of SSRs were mononucleotide followed by dinucleotide and trinucleotide repeats. Using Primer 3.0, primer pairs for each SSR were designed. These SSRs can be used for genetic map development and molecular breeding within *Lilium*.

## 4. Materials and methods

### 4.1. Plant Materials

Three purple-ovaried Asiatic lily cultivars (“Tiny Padhye”, “Black Eye”, and “Dixie Jazz”) and three green-ovaried Asiatic lily cultivars (“Dot.com”, “Pink Pixels”, and “Yellow Pixels”) (Figure S1) were used in this study. Plants were grown in a greenhouse at the Chinese Academy of Agricultural Sciences (Beijing, China). Ovary sampling was performed at three different developmental stages: stage 1 (S1, bud length of about 2.50 cm); stage 2 (S2, 3 days post-anthesis); stage 3 (S3, 9 days post-anthesis) (Figure 1). Peels were sampled from ten ovaries at each stage and pooled to improve sample homogeneity and decrease sampling bias. Three biological replicates were used for each sample. All samples were immediately frozen in liquid nitrogen and stored at −80 °C.

### 4.2. Measurement of Anthocyanin Content

Anthocyanins were extracted with a solvent mixture of trifluoroacetic acid, methanol, methane acid, and water (1:70:2:27, *v*:*v*:*v*:*v*). The extract solution of each sample was then analyzed using HPLC according to the methods of Cunja et al. [34].

### 4.3. cDNA Library Construction, Sequencing, and Transcriptome Assembly

Total RNA was extracted from ovary wall using an RNAprep pure Plant Kit (Tiangen Biotech Co., Ltd., Beijing, China) according to the manufacturer’s protocol. A total of six RNA-seq libraries (three biological replicates for each sample) were generated according to the manufacturer’s protocol of the ScriptSeq mRNA-Seq Library Preparation Kit (Epicentre Biotechnologies, Madison, WI, USA) and were sequenced to generate 150 paired-end raw reads on an Illumina Hiseq 4000 platform. After clean data were generated according to [35], transcriptome assembly was performed using Trinity software as previously described for de novo transcriptome assembly without a reference genome [36].

### 4.4. Functional Annotation and Classification

The assembled unigenes were searched against public databases, including the NCBI Non-Redundant Protein Sequences (NR) database, NCBI Nucleotide Sequences (NT) database, Protein Family (PFAM) database, Eukaryotic Ortholog Groups (KOG) database, Swiss-Prot protein database, Kyoto Encyclopedia of Genes and Genomes (KEGG) database, and Gene Ontology (GO) database. Gene Ontology (GO) enrichment analysis was performed on the differentially expressed genes (DEGs) using the GOseq R package [37].

### 4.5. Differentially Expressed Genes Analysis

The differentially expressed genes (DEGs) between green ovaries and purple ovaries were identified with the R package DESeq [38]. |log_2_ (foldchange)| > 1 and *q*-value < 0.01 were set as the thresholds for significantly different gene expression between the two samples. The heat maps showing expression profiles were generated based on the log_10_ (FPKM + 1) values using HemI (Heat map illustrator) with the default values [39].

### 4.6. Phylogenetic Analysis

A phylogenetic tree of selected R2R3-MYB transcription factors was constructed using MEGA 5 software by the neighbor-joining method with bootstrap analysis of 1000 replicates [40].

### 4.7. Quantitative Real-Time PCR Analyses

A total of 11 unigenes related to anthocyanin biosynthesis were chosen for quantitative real-time PCR (qPCR) analyses. qPCR analyses were performed using SYBR^®^ Premix Ex Taq™ II (Tli RNaseH Plus) (Takara, Dalian, China) and a Bio-Rad iQ5 Gradient Real Time PCR system with the following reaction conditions: one cycle of 95 °C for 30 s, followed by 40 cycles of 95 °C for 5 s and 60 °C for 30 s. The lily *Actin* gene was used as an internal control to normalize the expression data. Relative gene expression levels were calculated using the 2^−ΔΔ*C*t^ Method [41]. All primers used are listed in Table S3. Experiments were performed with three biological replicates and three technical replicates.

### 4.8. Effect of Shading Treatment on Pigment Accumulation and Gene Expression

To investigate the effect of shading treatment on pigment accumulation and gene transcription, 2.50 cm Asiatic lily “Tiny Padhye” flower buds were covered with silver paper. When ovary development reached stage 2, the paper was removed from the buds, and pigments and RNA were immediately extracted from the ovary wall. Plants exposed to light were used as the control group. Pigment measurement, RNA isolation, and qPCR were performed as described above.

### 4.9. Identification SSRs in ESTs and Primer Design

MISA (MIcroSAtellite; http://pgrc.ipk-gatersleben.de/misa) was used for microsatellite mining, and the minimum number of repeats used for selecting the SSRs was ten repeats for mononucleotide motifs and five repeats for dinucleotide, trinucleotide, tetranucleotide, pentanucleotide, and hexanucleotide motifs. Primer pairs for SSRs were designed using Primer 3.0 software (Premier Biosoft International, Palo Alto, CA, USA), with the following criteria: primer length ranging from 17 to 23 bp, melting temperature of 40–60 °C, GC content of 40%–60%, and PCR product size from 150 to 300 bp, and other parameter values as default.

## 5. Conclusions

In the present study, the transcriptomes of green ovaries (S1) and purple ovaries (S2) of the purple-ovaried cultivar “Tiny Padhye” were surveyed using the RNA-seq technology. Using this approach, ten putative anthocyanin structural genes and one *R2R3-MYB TF* were selected as the candidate genes most likely involved in determining the distribution of anthocyanins in Asiatic hybrid ovaries, and the expression level of these genes in green ovaries (S1) was much higher than purple ovaries (S2). In addition, shading treatment significantly decreased pigment accumulation by suppressing the expression of several unigenes related to anthocyanin biosynthesis in the ovaries of Asiatic “Tiny Padhye”. The transcriptome data from this study will facilitate our understanding of the molecular mechanisms of anthocyanin biosynthesis in the ovaries of Asiatic hybrid lilies.

## Figures and Tables

**Table 1:** Candidate anthocyanin structural genes and transcription factor genes in purple ovaries.

Gene	Protein	No. All *^a^*	No. Up *^b^*	No. Down *^c^*
*CHS*	Chalcone synthase	38	11	0
*CHI*	Chalcone isomerase	1	1	0
*F3H*	Flavanone 3-hydroxylase	2	1	0
*F3*′*H*	Flavonoid 3′-hydroxylase	4	1	0
*DFR*	Dihydroflavonol 4-reductase	3	1	0
*ANS*	Anthocyanidin synthase	3	0	0
*UFGT*	Anthocyanidin 3-*O*-glucosyltransfersae	5	3	0
*3RT*	anthocyanidin-3-glucoside rhamnosyltransferase	1	1	0
*R2R3-MYB*	R2R3-MYB transcription factors	28	8	0
*bHLH*	Basic helix–loop–helix transcription factors	19	8	11
*WD*	WD-repeat proteins	19	3	6

*^a^* The total number of uni-transcripts analyzed; *^b^* The number of uni-transcripts with expression significantly up-regulated in purple ovaries compared with green ovaries; *^c^* The number of uni-transcripts with expression significantly down-regulated in purple ovaries compared with green ovaries.

**Table 2:** Putative anthocyanin structural genes and transcription factor R2R3-MYBs identified among differentially expressed genes. Unigenes, showing significantly higher transcript levels in purple ovaries than in green ones, were highlighted in bold.

Annotation	Unigene ID	S1 FPKM	S2 FPKM
*CHS*	**c124520_g1**	**1.07**	**1255.60**
	**c114453_g1**	**1.91**	**1495.41**
	**c122851_g2**	**2.16**	**1996.29**
	**c124520_g2**	**2.15**	**2926.54**
	c201597_g1	1.17	106.51
	c115882_g3	0.96	614.86
	c122851_g1	0.00	342.93
	c115882_g2	0.95	375.32
	c81738_g1	0.00	12.03
	c114453_g2	0.06	3.35
	c124865_g1	0.07	1.22
*CHI*	**c123012_g2**	**93.54**	**743.77**
*F3H*	**c121630_g1**	**25.79**	**1134.48**
*F3'H*	**c113932_g1**	**25.64**	**1099.95**
*DFR*	**c124316_g1**	**8.43**	**5147.89**
*UFGT*	**c113956_g1**	**5.67**	**3286.62**
	c114752_g1	8.03	171.41
	c92724_g2	13.57	49.55
*3RT*	**c117809_g1**	**1.20**	**584.93**
*R2R3-MYB*	**c51332_g1**	**2.81**	**264.36**
	c72124_g1	0.00	1.75
	c116256_g1	2.26	10.81
	c36531_g1	2.47	7.92
	c121270_g3	42.98	256.59
	c96386_g1	65.52	182.05
	c116626_g1	2.21	5.60
	c104994_g2	0.00	16.16

**Table 3:** Statistics of the Simple Sequence Repeats (SSRs) identified in *Lilium* transcriptome.

Parameter	Value	Parameter	Value
SSR Identification		Distribution to Different Repeat Type Classes	
Total number of sequences examined	295,787	Mononucleotide	8163 (54.2%)
Total size of examined sequences (bp)	160,816,025	Dinucleotide	4446 (29.5%)
Total number of identified SSRs	15,048	Trinucleotide	2309 (15.3%)
Number of SSR containing sequences	13,710	Tetranucleotide	96 (0.6%)
Number of sequences containing more than one SSR	1201	Pentanucleotide	15 (0.1%)
Number of SSRs present in compound formation	444	Hexanucleotide	19 (0.1%)

## Supplementary Materials

Supplementary materials can be found at www.mdpi.com/1422-0067/17/11/1881/s1.
