# Supplementary material for: Transcriptome Analysis Identifies Key Candidate Genes Mediating Purple Ovary Coloration in Asiatic Hybrid Lilies
Source: Int J Mol Sci. 2016 Nov 20;17(11):1881. doi: 10.3390/ijms17111881 (PMC5133881; doi:10.3390/ijms17111881)
Supplement: Supplementary file 1 [file ijms-17-01881-s001.zip › ijms-151315-si- figure.pdf]

# Supplementary Materials: Transcriptome Analysis Identifies Key Candidate Genes Mediating P urple Ovary Coloration in Asiatic Hybrid Lilies

**Leifeng Xu, Panpan Yang, Suxia Yuan, Yayan Feng, Hua Xu, Yuwei Cao and Jun Ming**

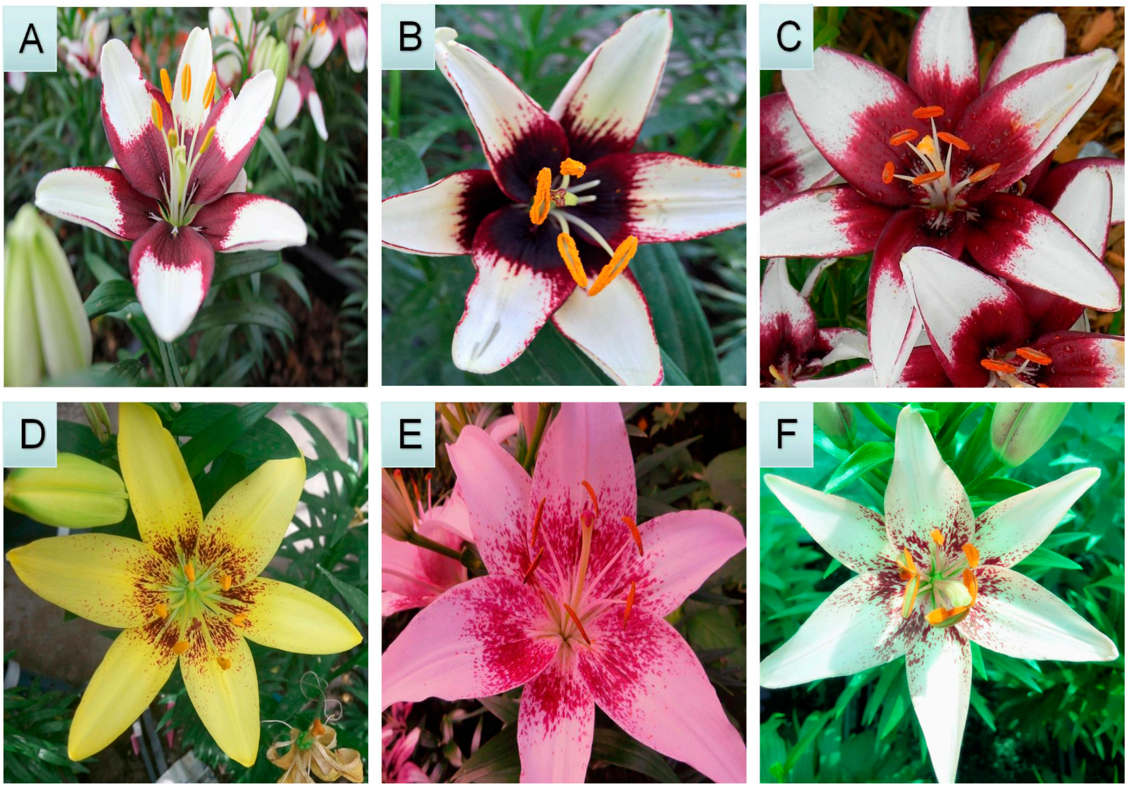


**Figure S1.** Flowers of six Asiatic hybrid lily cultivars: "Tiny Padhye" (**A**), "Black Eye" (**B**), "Dixie Jazz" (**C**), "Yellow Pixels" (**D**), "Dot.com" (**E**), and "Pink Pixels" (**F**).
